# Nuclear Factor Erythroid 2 Related Factor 2 Activator JC-5411 Inhibits Atherosclerosis Through Suppression of Inflammation and Regulation of Lipid Metabolism

**DOI:** 10.3389/fphar.2020.532568

**Published:** 2020-11-16

**Authors:** Xinhai Jiang, Yining Li, Weizhi Wang, Xiaowan Han, Jiangxue Han, Mingzhu Chen, Jing Zhang, Chenyin Wang, Shunwang Li, Jinque Luo, Xiao Wang, Yang Xu, Yanni Xu, Jingcai Cheng, Shuyi Si

**Affiliations:** ^1^ NHC Key Laboratory of Biotechnology of Antibiotics, National Center for New Microbial Drug Screening, Institute of Medicinal Biotechnology, Chinese Academy of Medical Sciences and Peking Union Medical College (CAMS and PUMC), Beijing, China; ^2^ JC (Wuxi) COMPANY, Inc., Wuxi, China

**Keywords:** Phenethyl isothiocyanate, JC-5411, nuclear factor erythroid 2-related factor 2, atherosclerosis, inflammation

## Abstract

Phenethyl isothiocyanate is widely present in cruciferous vegetables with multiple biological effects. Here we reported the antiatherogenic effects and the underlying mechanisms of JC-5411 (Phenethyl isothiocyanate formulation) *in vitro* and *in vivo*. Luciferase reporter assay showed that JC-5411 increased the activity of nuclear factor erythroid 2-related factor 2 (Nrf2) and antioxidant response element (ARE). JC-5411 treatment significantly increased the protein expression of Nrf2 and its downstream target gene hemeoxygenase 1 (HO-1) in liver of apolipoprotein E deficient (ApoE^−/−^) mice. Importantly, JC-5411 treatment significantly reduced atherosclerotic plaque area in both en face aorta and aortic sinus when compared with model group in WD induced ApoE^−/−^ mice. JC-5411 obviously decreased proinflammatory factors’ levels in serum of ApoE^−/−^ mice, LPS stimulated macrophages and TNFα induced endothelial cells, respectively. JC-5411 significantly decreased the levels of total cholesterol (TC) and triglyceride (TG) in both serum and liver of ApoE^−/−^ mice and hyperlipidemic golden hamsters. Mechanism studies showed that JC-5411 exerted anti-inflammatory effect through activating Nrf2 signaling and inhibiting NF-κB and NLRP3 inflammasome pathway. JC-5411 exerted regulating lipid metabolism effect through increasing cholesterol transfer proteins (ABCA1 and LDLR) expression, regulating fatty acids synthesis related genes (p-ACC, SCD1 and FAS), and increasing fatty acids β-oxidation (CPT1A) *in vivo*. Furthermore, JC-5411 treatment had a favorable antioxidant effect in ApoE^−/−^ mice by increasing the antioxidant related genes expression. Taken together, we conclude that JC-5411 as a Nrf2 activator has anti-inflammatory, rebalancing lipid metabolism, and antioxidant effects, which makes it as a potential therapeutic agent against atherosclerosis.

## Introduction

Atherosclerosis, the underlying pathological basis for numerous cardiovascular diseases, is a chronic disease with complex pathogenesis (Wolf and Ley, 2019). It is well established that inflammation, lipid deposition, and oxidative stress are all involved in the initiation and progression of atherosclerosis (Libby et al., 2016; Wu et al., 2017a; Libby et al., 2011). Inflammation is an important driver and one major risk factor in the progression of atherosclerosis. Inflammation has been shown to lead to endothelial dysfunction and results in the increasing monocyte adhesion and formation of foam cells, leading to lipid accumulation in the artery wall and ultimately to atherosclerosis (Mimura and Itoh, 2015; Chen and Frishman, 2016; Gimbrone and García-Cardeña, 2016; Grebe et al., 2018). Additionally, there is a strong associative link between high level of serum lipids and an increased risk of atherosclerosis (Libby et al., 2011). It has been observed that high levels of serum lipid lead to lipid accumulation in the artery wall, which accelerates the progression of atherosclerosis. A further contributor to the deterioration of the vessel and the acceleration of atherosclerotic plaques are uncontrolled redox processes, as observed in the presence of reactive oxygen species (ROS) (Forstermann et al., 2017). Therefore, alleviating inflammation, improving lipid metabolism, and limiting oxidative stress all have the potential for anti-atherosclerosis treatment strategies (Libby et al., 2011; Hansson et al., 2006; Kattoor et al., 2017).

Nuclear factor erythroid 2-related factor 2 (Nrf2), regarded as a redox-sensitive switch in cells, is a member of the Cap’n’Collar (CNC)-bZIP transcription factor family (Cuadrado et al., 2019; Yamamoto et al., 2018). Nrf2 binds to its natural repressor Kelch like ECH-associated protein 1 (Keap1) and degrades rapidly through proteasomal degradation under basal conditions. However, Nrf2 can translocate from Keap1, and subsequently binds to the antioxidant response element (ARE) in the nucleus (Mimura and Itoh, 2015). This in turn regulates downstream gene expression that includes hemeoxygenase 1 (HO-1), glutathione (GSH) related genes and superoxide dismutase 1 (SOD1) upon oxidative stress (Tonelli et al., 2018). Many studies have shown that Nrf2 signaling modulates physiological processes associated with the progression of atherosclerosis, such as redox regulation, inflammation and lipid homeostasis (Mimura and Itoh, 2015). Moreover, several studies suggest that upregulating Nrf2 signaling may have a beneficial effect on the prevention of atherosclerosis (Jakobs et al., 2017; Zhu et al., 2019; Zhang et al., 2016; Zakkar et al., 2009). It has been reported that Nrf2 signaling activators such as hydrogen sulfide, and isothiocyanates exert anti-inflammatory and antioxidant effects *in vitro* and *in vivo* (Huang et al., 2013; Aboonabi and Singh, 2015; Xie et al., 2016).

Phenethyl isothiocyanate (PEITC) is widely present in cruciferous vegetables with multiple biological effects (Dayalan Naidu et al., 2018). PEITC has been shown to exert anti-inflammatory effects in endotheliocytes through activation of the Nrf2 signaling pathway. This activation subsequently leads to an increase in the expression of Nrf2 and its downstream target gene HO-1 (Huang et al., 2013). But the direct anti-atherosclerosis activity of PEITC *in vivo* is unknown. In this study we utilized apolipoprotein E-deficient (ApoE^−/−^) atherosclerosis mouse models to explore whether JC-5411 (PEITC formulation) has anti-atherosclerosis activity *in vivo*, and to ascertain the underlying mechanism of its action. According to our data, we report JC-5411 exhibits antiatherogenic activities through attenuation of inflammation, improvement of lipid metabolism and increase of antioxidant activity.

## Materials and Methods

### Reagents

JC-5411 used in animal experiments is a formulated PEITC capsule provided by JC (Wuxi) COMPANY, Inc., Wuxi, China. JC-5411 used in *in vitro* assays is a formulated PEITC liquid provided by provided by JC (Wuxi) COMPANY, Inc., Wuxi, China. In this article, they are collectively called JC-5411.

### Cell Culture

Human Umbilical Vein Endothelial Cells (HUVEC) (PromoCell, Heidelberg, Germany) were cultured in endothelial cell growth medium (PromoCell, Heidelberg, Germany). THP-1 cells (ATCC, Rockville, MD) were grown in RPMI 1640 (Thermo Fisher Scientific, Waltham, MA) with 10% Fetal Bovine Serum (FBS) (Thermo Fisher Scientific, Waltham, MA). HepG2 cells (ATCC, Rockville, MD) were cultured in DMEM (Thermo Fisher Scientific, Waltham, MA) with 10% FBS. J774A.1 mouse macrophage cell line (ATCC, Rockville, MD) was cultured in RPMI 1640 with 10% FBS. All cells were cultured at 37°C with 5% CO_2_ in a cell incubator.

### Transfection and Luciferase Assay

HepG2 cells were co-transfected with pGL4.73 (luc2P-ARE-Hygro, Promage, Madison, WI) and pGL4.75 (hRluc/CMV, Promage, Madison, WI) (mass ratio 1:100, 10 μg total DNA for a 100-mm dish of cells) using Lipofectamine 2000 (Thermo Fisher Scientific, Waltham, MA) in a 96-well plate for 6 h. JC-5411 were diluted to different concentration (1.25, 2.5, 5 and 10 μM) with DMEM containing 10% FBS and added to a 96-well plate (200 μl per well) for 24 h. The luciferase reporter gene activity was then measured using Dual-Glo^®^ Luciferase Assay System (Promage, Madison, WI).

pNLF1-NRF2 (CMV/neo, Promage, Madison, WI) and pKEAP1 (Promage, Madison, WI) (mass ratio 1:100, 10 μg total DNA for a 100-mm dish of cells) were co-transfected into HepG_2_ cells using Lipofectamine 2000. After incubating with JC-5411 (active pharmaceutical ingredient) for 24 h, the reporter gene activity was measured using Nano-Glo^®^ luciferase Assay System (Promage, Madison, WI).

### Western Blot Analysis

The fresh liver tissues were homogenized and the total proteins from tissues or cells were extracted using RIPA buffer (Applygen Technologies, Beijing, China) supplemented with 1 mmol/L phenylmethanesulfonyl fluoride (PMSF) (Applygen Technologies, Beijing, China). The protein concentrations were determined with bicinchoninic acid (BCA) Protein Assay Kits (Thermo Fisher Scientific, Waltham, MA), and then separated by SDS-PAGE gels.

Target proteins were detected by Western blot with the corresponding antibodies. The antibodies used in this study included Nrf2 (Abcam, cambridge, United Kingdom), HO-1 (Abcam, cambridge, United Kingdom), *β*-actin (Abcam, cambridge, United Kingdom), ABCA1 (Novus Biologicals, Colorado, United States), LDLR (Abcam, Cambridge, United Kingdom), CPT1A (Abcam, cambridge, United Kingdom), SCD1 (Abcam, cambridge, United Kingdom), ACC (Cell Signaling Technology, Danvers, MA), p-ACC (Cell Signaling Technology, Danvers, MA), NLRP3 (Abcam, cambridge, United Kingdom), pro-Caspase-1 (Abcam, cambridge, United Kingdom), IL-1β (Abcam, cambridge, United Kingdom), p65 (Cell Signaling Technology, Danvers, MA), p-p65 (Abcam, cambridge, United Kingdom) ICAM-1 (Abcam, cambridge, United Kingdom), VCAM-1 (Abcam, cambridge, United Kingdom), PPARα (Abcam, cambridge, United Kingdom), LXRα (Abcam, cambridge, United Kingdom), SREBP-1 (Abcam, cambridge, United Kingdom), FAS (Abcam, cambridge, United Kingdom) and the corresponding secondary anti-rabbit and anti-mouse IgG antibodies (Cell Signaling Technology, Danvers, MA). All relative protein levels of target genes were normalized to *β*-actin. The intensities of protein band were analyzed by NIH Image J software.

### RNA Isolation and Real-Time Quantitative PCR (RT-qPCR)

The fresh liver tissues were homogenized, total RNA was extracted using QIAGEN RNeasy Mini kit (Qiagen, Hilden, Germany), reverse transcribed using TransScript One-Step gDNA Removal, and cDNA synthesized using Synthesis SuperMix (Transgen Biotech, Beijing, China). Real-time quantitative PCR (RT-qPCR) assay was performed using FastStart Universal SYBR Green Master (Roche) and corresponding primers with FTC-3000 Real-Time Quantitative Thermal Cycler (Funglyn Biotech Inc, Richmond Hill, Canada). Relative mRNA expression of target genes was normalized to GAPDH and the quantification result was calculated using the ^△△^Ct method.

The sequences of the primers used were as follows: mouse Nrf2 (forward: 5′-CTC​CGT​GGA​GTC​TTC​CAT​TTA​C-3′, reverse: 5′- GCA​CTA​TCT​AGC​TCC​TCC​ATT​TC-3′); mouse HO-1 (forward: 5′- GTA​CAC​ATC​CAA​GCC​GAG​AA-3′, reverse: 5′-TGG​TAC​AAG​GAA​GCC​ATC​AC -3′); mouse NQO1 (forward: 5′-GAG​AAG​AGC​CCT​GAT​TGT​ACT​G -3′, reverse: 5′- ACC​TCC​CAT​CCT​CTC​TTC​TT-3′); mouse GCLC (forward: 5′- CAT​CGA​CCT​GAC​CAT​CGA​TAA​G -3′, reverse: 5′- AGG​GTG​AGT​GGG​TCT​CTA​ATA​A -3′); mouse GCLM (forward: 5′- CAG​CCT​TAC​TGG​GAG​GAA​TTA​G -3′, reverse: 5′- GCT​CCA​ACT​GTG​TCT​TGT​CT -3′); mouse IL-6 (forward: 5′- CAG​CCT​TAC​TGG​GAG​GAA​TTA​G -3′, reverse: 5′- GCT​CCA​ACT​GTG​TCT​TGT​CT -3′); mouse IL-1β (forward: 5′- CCA​CCT​CAA​TGG​ACA​GAA​TAT​CA -3′, reverse: 5′- CCC​AAG​GCC​ACA​GGT​ATT​T -3′); mouse TNFα (forward: 5′- CTT​CCA​TCC​AGT​TGC​CTT​CT -3′, reverse: 5′- CTC​CGA​CTT​GTG​AAG​TGG​TAT​AG -3′); human Nrf2 (forward: 5′- TGA​TTC​TGA​CTC​CGG​CAT​TT -3′, reverse: 5′- GCC​AAG​TAG​TGT​GTC​TCC​ATA​G -3′); human HO-1 (forward: 5′- ACC​AAG​TTC​AAG​CAG​CTC​TAC -3′, reverse: 5′- GCA​GTC​TTG​GCC​TCT​TCT​ATC -3′); human GCLC (forward: 5′- CCC​AAA​CCA​TCC​TAC​CCT​TT -3′, reverse: 5′- CAT​GTT​GGC​CTC​AAC​TGT​ATT​G -3′); human GCLM (forward: 5′- GAG​TTG​CAC​AGC​TGG​ATT​CT -3′, reverse: 5′- CCT​CCC​AGT​AAG​GCT​GTA​AAT​G -3′); human NQO1 (forward: 5′- GGG​ATG​AGA​CAC​CAC​TGT​ATT​T -3′, reverse: 5′- TCT​CCT​CAT​CCT​GTA​CCT​CTT​T -3′).

### Monocyte Adhesion to Human Umbilical Vein Endothelial Cells

HUVECs were cultured in 6-well plates and incubated with vehicle (0.1% DMSO) or JC-5411 (1 and 5 μM) in an incubator for 18 h. Human tumor necrosis factor alpha (TNFα, final concentration 10 ng/ml; R&D Systems, Minneapolis, MN) was then added to the culture, and the cells were incubated for another 6 h followed by the addition of approximately 7×10^6^ THP-1 cells to the culture and co-cultured for 30 min (Xu et al., 2017). The cells were gently washed with endothelial cell growth medium twice and examined under a microscope (Leica CM1950; Wetzlar and Mannheim, Germany).

### siRNA Transfection

HUVECs were transfected with the negative control siRNA (siNC final concentration 50 nM; Santa Cruz Biotechnology, Santa Cruz, CA) or Nrf2 siRNA (final concentration 50 nM; Santa Cruz Biotechnology, Santa Cruz, CA) in opti-MEM (Thermo Fisher Scientific, Waltham, MA), with Lipofectamine RNAi^MAX^ (Thermo Fisher Scientific, Waltham, MA). Six hours later, the medium was replaced with endothelial cell growth medium (PromoCell GmbH, Heidelberg, Germany), and cells were incubated for another 24 h before treating with or without JC-5411 for another 18 h followed by adding TNFα (final concentration 10 ng/ml) to the culture, and continue to incubate for 6 h. The monocyte adhesion assays or western blot and were then performed.

To established oxidative stress model in HUVECs, after siNC or Nrf2 siRNA was transfected, cells was incubated with or without JC-5411 (5 μM) for 18 h, then H_2_O_2_ (200 μM) was added for another 6 h, the total mRNA was extracted for RNA analysis.

Macrophage J774.1 cells were transfected with siNC or Nrf2 siRNA as described above, then cells were treated with lipopolysaccharide (LPS) (1 μg/ml) (Beyotime Biotechnology, Beijing, China) with or without JC-5411 (5 μM) for another 24 h, western blot or elisa assays were then performed.

### Anti-Atherosclerosis Analysis of JC-5411 Treatment in ApoE^−/−^ Mice

Eight-week-old male ApoE^−/−^ mice were purchased from Beijing VitalRiver Laboratory Animal Technology Co., Ltd. (Beijing, China). To establish the model of atherosclerosis, ApoE^−/−^ mice were fed with a Western Diet (WD) (TP26300 (21% fat, 0.2% cholesterol, 49.1% carbohydrate, 19.8% protein), Trophic Animal Feed High-tech Co., Ltd., Nantong, China) while JC-5411 (60 mg/kg, in 0.5% carboxymethylcellulose-Na (CMC-Na) or 0.5% CMC-Na (model group) were given by gavage paralleled with WD feeding. Rosuvastatin (10 mg/kg; AstraZeneca, London, United Kingdom, Lot number: 134160) was selected as positive control and was given to the mice once a day in 0.5% CMC-Na. Considering the pharmacokinetics characteristics of JC-5411 (Ji et al., 2005), JC-5411 (60 mg/kg) was intragastrically administered twice a day for 10 weeks. ApoE^−/−^ mice in the control group were kept on a normal laboratory diet (ND, #Co60, SPF; Beijing Biotechnology Co., Ltd.) and intragastrically administered 0.5% CMC-Na. Body weight was monitored weekly. All animal experiments were approved by the Institutional Animal Care and Use Committee of the Institute of Medicinal Biotechnology Institute and performed in accordance with the regulations, and the ethical review number is IMB-20180320-D1.

At the end of administration, blood samples were taken from the retro-orbital plexus under fasted conditions before mice were euthanized. Serum levels of TC high density lipoprotein cholesterol (HDL-C), low density lipoprotein cholesterol (LDL-C) and triglyceride (TG) were measured using Automatic biochemical analyzer (Hitachi 71800, Chiyoda, Japan) and the commercial available Assay Kits (BIOSINO Bio-Technology and Science, Beijing, China). Serum levels of TNFα, Interleukin-1β (IL-1β), intercellular adhesion molecule-1 (ICAM-1) and vascular cell adhesion molecule-1 (VCAM-1) were determined, respectively, by enzyme linked immunosorbent assay method using the corresponding Elisa Kits (SBJ bio, Nanjing, China).

Whole aortas from ApoE^−/−^ mice were isolated carefully and fixed in 4% formalin. Aortas were stained with Oil Red O (ORO) (Sigma-Aldrich, St. Louis, MO) for 30 min, and the images of the open luminal surface were captured with a digital camera (Sony). The fixed heart with upper aortic root was embedded in optimum cutting temperature compound (Sakura Finetek, Torrance, CA) and then sectioned into serial 7 μm cryosections of aortic sinus cross sections on a cryostat (Leica, Microsystems, Wetzlar, Germany). To analyze the areas of atherosclerotic lesion in aortic sinus cross, ORO and hematoxylin and eosin (H&E) (Beyotime Biotechnology, Beijing, China) were used to stain the cryosections, respectively. Both lesion areas of the stained aortic sinus cross sections and aortas were analyzed with NIH Image J software.

The livers were collected and frozen in liquid nitrogen promptly before storing at −80°C for preparations of mRNA, protein or others as indicated. TG and TC in liver were extracted and measured with corresponding Assay Kit (Applygen Technologies, Beijing, China), and the contents of TG and TC were finally expressed as μmol per gram protein.

The SOD activity and Malonaldehyde (MDA) level in the liver were determined with a SOD Activity Assay Kit (Beyotime Biotechnology, Beijing, China) and an MDA Assay Kit (Beyotime Biotechnology, Beijing, China), respectively.

The superoxide (O_2_^−^) production in atherosclerotic lesions was detected by dihydroethidium (Thermo Fisher Scientific, Waltham, MA) according to the manufacturer’s instructions. Briefly, the aortic sinus cross sections were incubated with dihydroethidium for 30 min, and then washed with phosphate buffer saline for three times. The fluorescence was detected with High Content Analysis System (Operetta CLS™, PerkinElmer, Fremont, CA), and the positive staining area was analyzed using NIH Image J software.

### Hyperlipidemia Golden Hamsters Treated With JC-5411

Eight-week-old male golden hamsters were purchased from Beijing VitalRiver Laboratory Animal Technology Co, Ltd. The hamsters were given a standard chow diet in the negative control group, or were fed on high fat diet (HFD, TP4H100, 20% fat, 40% fructose, 0.25% cholesterol, Trophic Animal Feed High-tech Co., Ltd., Nantong, China) for a week to establish hyperlipidemia disease model. Animals fed on HFD were further randomly divided into four groups, and were intragastrically treated with JC-5411 in 0.5% CMC-Na at dose of 20 mg/kg, 40 mg/kg, 60 mg/kg, or vehicle, twice a day for 3 weeks, respectively. The negative control group were continuously fed on ND and 0.5% CMC-Na for 3 weeks The hamsters were fasted overnight, and the blood and liver samples were collected. TG, TC, HDL-C and LDL-C level in serum as well as liver TG and TC were determined the same was as described in section of ApoE^−/−^ mice treatment. All animal experiments were approved by the Institutional Animal Care and Use Committee of the Institute of Medicinal Biotechnology Institute and performed in accordance with the regulations, and the ethical review number is IMB-20180913-D3.

### Immunofluorescence Staining

Cells and aortic sinus cross sections were treated as described above. Then cells and aortic sinus cross sections were fixed in 4% formalin for 30 min, then incubated with antibodies at 4°C overnight. After washed with phosphate buffer saline for three times, appropriate secondary antibodies were used for 30 min at temperature. Then nucleus was stained with 4’,6-diamidino-2-phenylindole (DAPI, Thermo Fisher Scientific, Waltham, MA). The fluorescence was detected and analysis with High Content Analysis System (Operetta CLS™, PerkinElmer, Fremont, CA).

### Immunohistochemical Staining

Livers from both ApoE^−/−^ mice and golden hamsters were fixed in 4% formalin overnight and stored in 20% sucrose at 4^o^C. The paraffin-embedded and optimum cutting temperature-embedded sections were then prepared for H&E and ORO staining, respectively. The interested images were captured using Leica Q550cw Graphic Analysis System (Leica Microsystems, Wetzlar, Germany).

### Fast Protein Liquid Chromatography (FPLC)

Total 300 μl serum from six different ApoE^−/−^ mice (50 μl per mice) per group was filtrated with Superose six columns (GE Healthcare Europe GmbH, Munich, Germany) with a speed of 0.2 ml per min buffer with ÄKTA explorer analysis system (GE Healthcare Europe GmbH, Munich, Germany). The TC level in each fraction was measured using the cholesterol assay kit mentioned above.

### Statistical Analysis

All the data were analyzed and presented as mean ± standard error of the mean (SEM). Student’s *t* test or one-way ANOVA analysis was conducted using GraphPad Prism eight software. A *p* < 0.05 was regarded as statistically significant.

## Results

### JC-5411 Increased Nuclear Factor Erythroid 2-Related Factor 2 and ARE Luciferase Reporter Gene Activity

**FIGURE 1:**
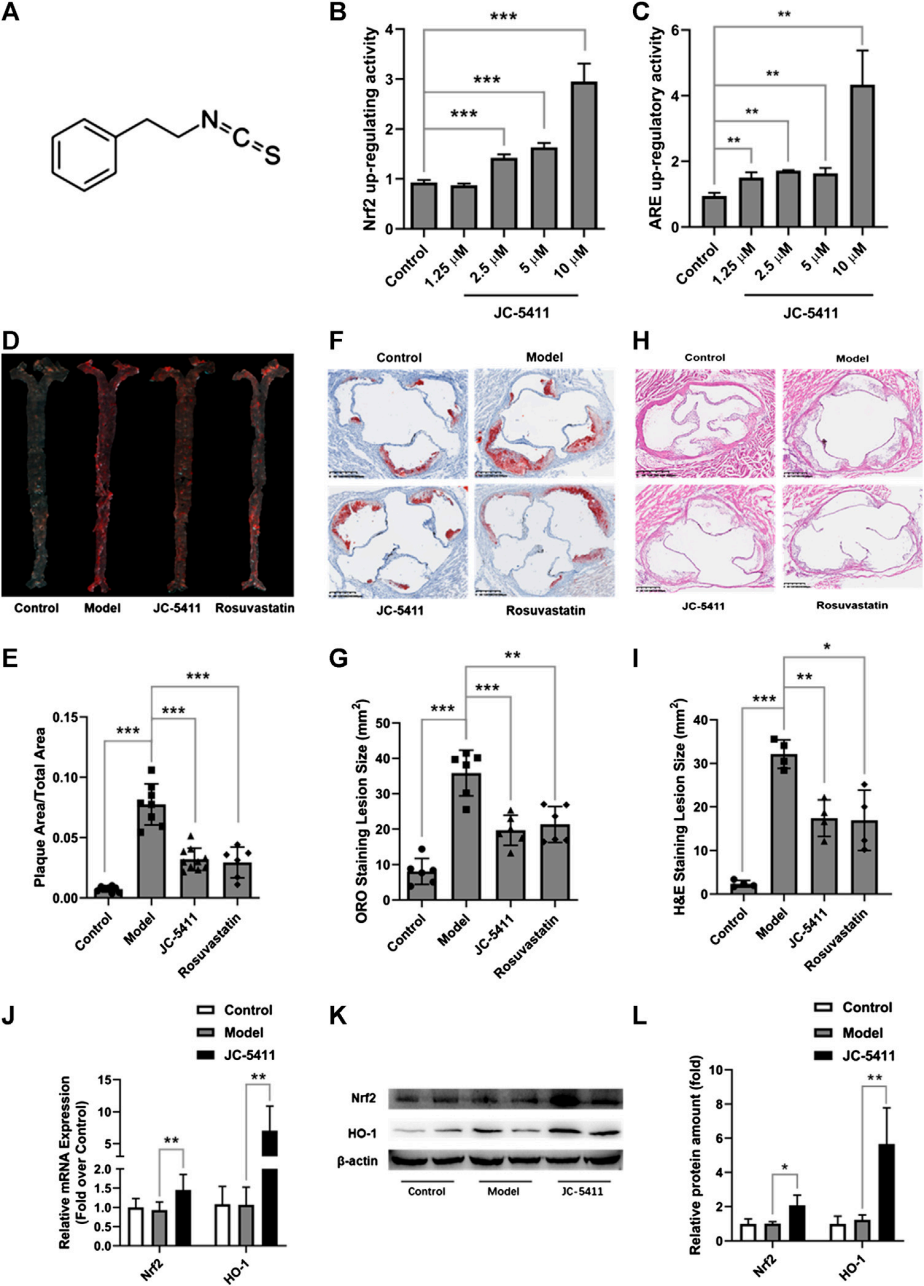
JC-5411 was a Nrf2 signaling activator and exerted anti-atherosclerosis effect in WD induced ApoE^−/−^ mice. **(A)** The chemical structure of JC-5411. **(B)** Increase in luciferase reporter gene activity of luc2P-ARE-Hygro and hRluc/CMV (Fold over control) by JC-5411. HepG_2_ cells were co-transfected with luc2P-ARE-Hygro and hRluc/CMV. After transfection, cells were treated with JC-5411 (1.25, 2.5, 5, and 10 μM) for 24 h, and the reporter activity was determined as described in the related section of “Method of Methods.” **(C)** Increase in luciferase reporter gene activity of pNLF1-NRF2 and pKEAP1 (Fold over control) by JC-5411. HepG_2_ cells were co-transfected with pNLF1-NRF2 and pKEAP1 24h, the transfected cells were then treated with JC-5411 (1.25, 2.5, 5 and 10 μM) for 24 h. **(B, C)** Student’s *t* test, * **p < 0.01, ***p < 0.001, *n* = 4. **(D, E)** Eight-week-old male ApoE^−/−^ mice were fed with a standard chow diet and given 0.5% CMC-Na (control) or WD and administered with 0.5% CMC-Na (model group) or WD and given JC-5411 (60 mg/kg) or rosuvastatin (positive control) for ten weeks. Respective images of aortas stained **(D)** and quantified **(E)** by ORO (plaque area/total area) of each group. One-way ANOVA analysis was performed: **p < 0.01, ***p < 0.001, *n* = 6–10. **(F, G)** Respective images of aortic sinus cross sections stained and quantified by ORO (lesion area size) of each group. One-way ANOVA analysis was performed, **p < 0.01, ***p < 0.001, *n* = 6. **(H, I)** Respective images of aortic sinus cross sections stained and quantified by H&E staining (lesion area size) of each group. One-way ANOVA analysis was performed, *p < 0.05, **p < 0.01, ***p < 0.001, *n* = 4. **(J–L)** The mRNA level was determined using RT-qPCR **(J)**, and the protein level of Nrf2 and HO-1 in the liver from ApoE^−/−^ mice described above were measured by western blot **(K, L)** Student’s *t* test was performed, *p < 0.05, ** *p* < 0.01, *n* = 4.

The structure of JC-5411 is shown in Figure 1A, and it is reported to be a Nrf2 activator (Dayalan Naidu et al., 2018). To confirm the effect of IC-5411 on the Nrf2-ARE signal pathway, pGL4.73 (luc2P-ARE-Hygro) and pGL4.75 (hRluc/CMV) or pNLF1-NRF2 (CMV/neo) and pKEAP1 were co-transfected into HepG_2_ cells as described in the methods section. Our results showed that JC-5411 increased the luciferase reporter gene activity of both Nrf2 and ARE significantly in a dose-dependent manner (Figure 1B, **C**), indicating that JC-5411 can indeed activate the Nrf2 signal pathway *in vitro*.

### JC-5411 Reduced Atherosclerotic Lesions in ApoE^−/−^ Mice

Upregulating Nrf2 signaling may reduce the risk of atherosclerosis, and we therefore speculate and examine whether JC-5411 has the potential to decrease atherosclerotic lesions in ApoE^−/−^ mice. ApoE^−/−^ mice fed on WD were treated with JC-5411 for 10 weeks and the subsequent disease lesions were quantitatively analyzed. As shown in Figures 1D, E, mice treated with JC-5411 exhibited lower plaque ratios (plaque area/total area) along the en face aorta than that of the mice in model group. JC-5411 treatment also resulted in decreased plaque area observed in ORO stained aortic root sections when compared to that of model group (Figures 1F, G). The H&E stain of aortic root sections also shows the same trend (Figures 1H, I). Moreover, as mentioned above, JC-5411 was found to increase Nrf2 luciferase reporter expression *in vitro*. To further confirm it acts as an activator of Nrf2 signaling, the expression of Nrf2 in livers was determined by RT-qPCR and western blot, respectively. The results showed that JC-5411 increases Nrf2 expression at both mRNA and protein level in the livers of ApoE^−/−^ mice (Figures 1J–L), demonstrating that JC-5411 activates Nrf2 signaling *in vivo*.

Collectively, our data suggest that JC-5411 can reduce atherosclerotic lesions in WD induced ApoE^−/−^ mice.

### JC-5411 Decreased Serum Proinflammatory Cytokines’ Levels in ApoE^−/−^ Mice

**FIGURE 2:**
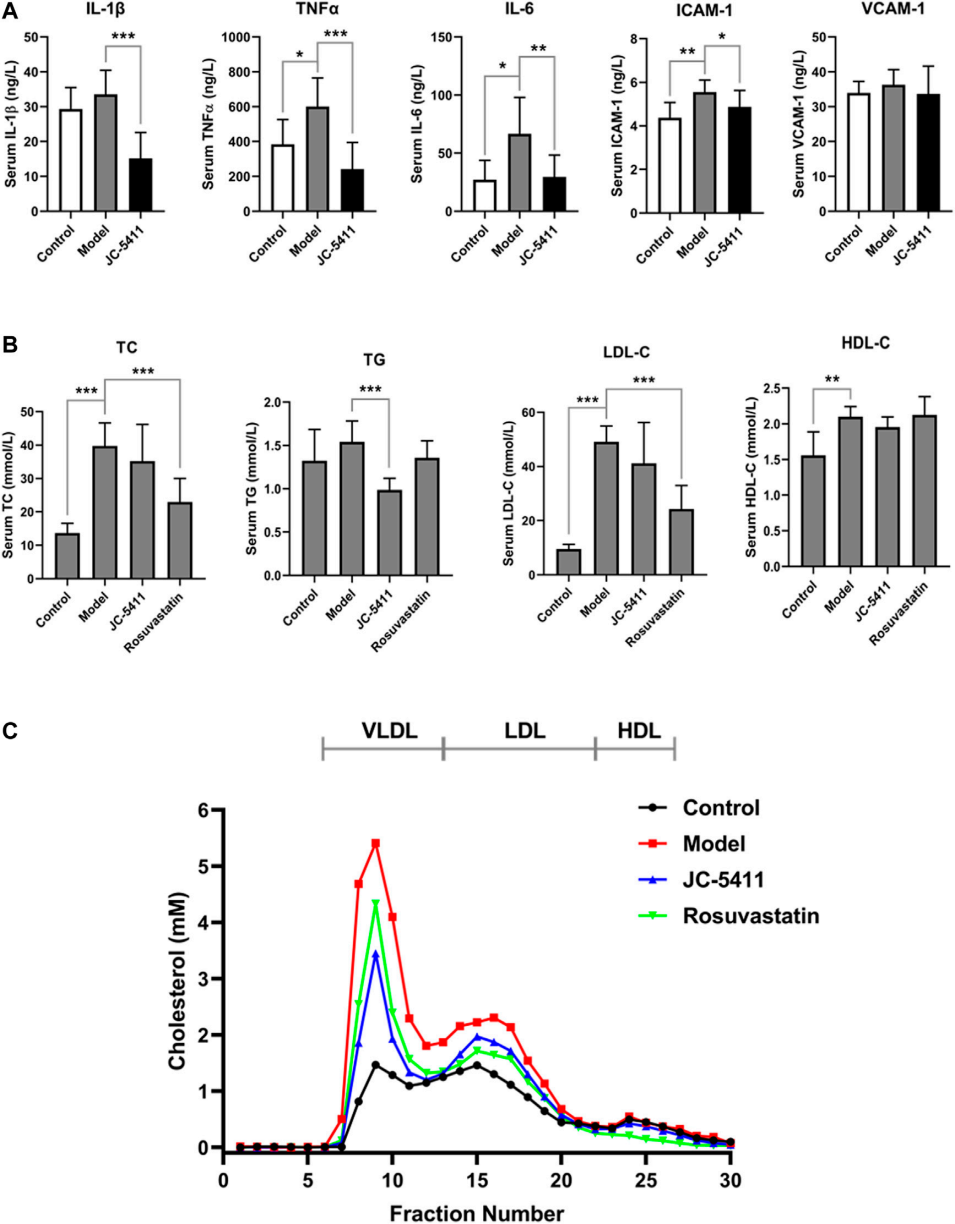
JC-5411 decreased proinflammatory cytokines and lipid level in the serum of ApoE^−/−^ mice. **(A)** Serum levels of TNFα, IL-1β, IL-6, ICAM-1 and VCAM-1 in the serum of ApoE^−/−^ mice were determined by enzyme linked immunosorbent assay. *n* = 8 per group. **(B)** Serum TC, TG, LDL-C and HDL-C level in ApoE^−/−^ mice. **(A, B)** One-way ANOVA was performed *p < 0.05, **p < 0.01, ***p < 0.001, *n* = 6–10. **(C)** FPLC assay results from serum of ApoE^−/−^ mice. Total 300 μl serum from six different mice (50 μl per mice) per group was filtrated with Superose six columns, and the cholesterol level in each fraction was measured using an assay kit.

Inflammation plays a crucial role in the development of atherosclerosis. To explore the anti-inflammatory effects of JC-5411 *in vivo*, serum levels of proinflammatory cytokines including TNFα, IL-1β and IL-6, VCAM-1 and ICAM-1 were examined using an enzyme-linked immunosorbent assay. As shown in Figure 2A, JC-5411 treatment significantly decreased the serum TNFα, IL-1β, IL-6, and ICAM-1 levels when compared with the model group. Furthermore, this is corroborated by the paralleled decreases in the mRNA level of TNFα, IL-1β and IL-6 in livers measured by RT-qPCR (Supplementary Figure S1A).

### JC-5411 Decreased Serum TG and VLDL Levels in ApoE^−/−^ Mice

In addition, serum lipid levels were determined as described in the methods. As shown in Figure 2B, serum TG level of JC-5411 treatment group was significantly lower than that of model group. Serum TC and LDL-C levels in JC-5411 treatment group were lower than those observed in the model group but with no statistically significant differences, while HDL-C levels remained unchanged (Figure 2B). And there is no change in the body weight of JC-5411 treated ApoE^−/−^ mice when compared with that in model group (Supplementary Figure S1B).

To further separate the lipoprotein classes in the serum of ApoE^−/−^ mice properly, FPLC was performed and the TC level in each fraction was detected. As shown in Figure 2C, when compared with model group, JC-5411-treatment decreased the serum VLDL (very low-density lipoprotein) level remarkably, and decreased LDL-C level in some degree.

### JC-5411 attenuated inflammatory activity via Nrf2 signaling in LPS-treated macrophage J774A.1 cell and ApoE^−/−^ mice

**FIGURE 3:**
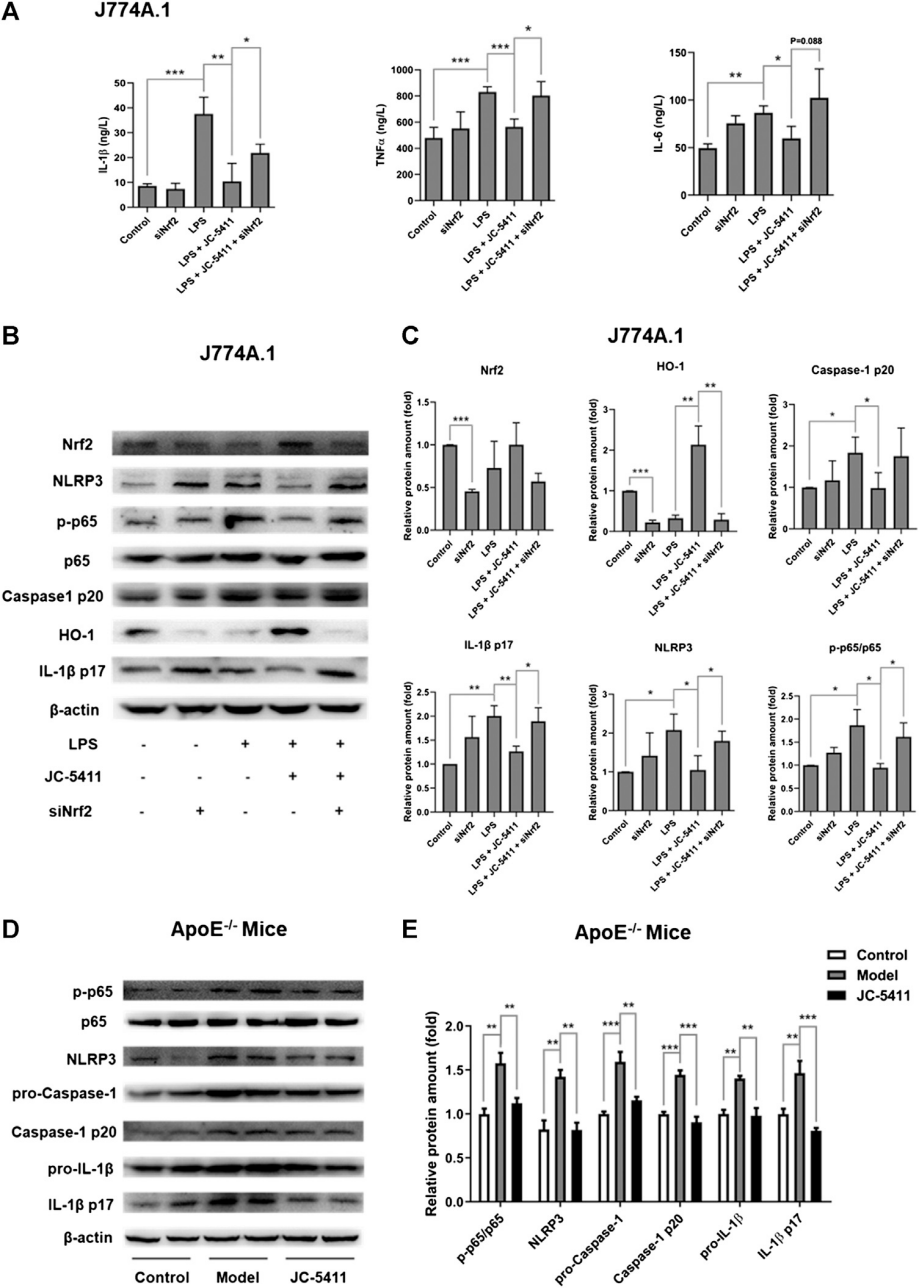
JC-5411 exerted anti-inflammatory effect via Nrf2 signaling both in LPS stimulated macrophages and in liver of ApoE−/− mice **(A)** The cytokines level in cell supernatants were detected by Elisa in LPS stimulated macrophage J774A.1 cell. One-way ANOVA analysis was performed, *p < 0.05, **p < 0.01, ***p < 0.001, *n* = 3. **(B, C)** Macrophage J774A.1 cells were treated as described in “Materials and Methods”. The protein level of Nrf2, HO-1, NLRP3, P65, p-P65, caspase-1 p20, IL-1β p17 was detected by Western blotting. All protein bands were normalized by *β*-actin and expressed as a fold of control. One-way ANOVA analysis was performed, *p < 0.05, **p < 0.01, *n* = 3. **(D, E)** Western blotting in liver of ApoE^−/−^ mice was conducted using antibodies against p-p65, p65, NLRP3, pro-Caspase-1, Caspase-1 p20, pro-IL-1β and IL-1β p17. All protein bands were normalized by *β*-actin and expressed as a fold of control. One-way ANOVA analysis was performed, **p < 0.01, ***p < 0.001, *n* = 4 per group.

The inflammatory state in macrophage is an initial event at the onset of atherosclerosis (Bäck et al., 2019). JC-5411 could significantly decrease serum proinflammatory cytokines’ level in WD induced ApoE^−/−^ mice, then we wondered whether it had anti-inflammatory effect in LPS-treated macrophage J774A.1 cell. LPS induced macrophage J774A.1 cell was used to establish the cell inflammation model as described in the methods. The results showed that LPS stimulation significantly increased proinflammatory cytokine’ level such as TNFα, IL-1β and IL-6 in the cell supernatants of macrophage J774A.1 cells (Figure 3A), while JC-5411 significantly reduced these increased cytokine levels (Figure 3A). Furthermore, this reduced effect of JC-5411 on the cytokines could be obviously reversed when *nrf2* knockdown, which indicated that the anti-inflammatory of JC-5411 in LPS induced macrophage was dependent on Nrf2 (Figure 3A).

After confirming the anti-inflammatory effects of JC-5411 in macrophages, we wondered how JC-5411 played anti-inflammatory effect *in vitro* and *in vivo*. Recent studies have indicated that the NOD-like receptor family pyrin domain containing 3 (NLRP3) inflammasome mediated inflammation plays a key role in the progression of atherosclerosis (Grebe et al., 2018; Hoseini et al., 2018; Baldrighi et al., 2017). The process of the NLRP3 inflammasome activation includes priming and NLRP3 activation stages. Initially, The nuclear factor κB (NF-κB) signaling is activated and the expression of NLRP3 and pro-IL-1β increases, which subsequently triggers the assembly of the inflammasome leading to the release of mature IL-1β (Grebe et al., 2018; Prochnicki and Latz, 2017; Bauernfeind et al., 2009). Therefore, we investigated whether JC-5411 mediated anti-inflammatory effects are related to NLRP3 inflammasome pathway. Our results showed that after treated with LPS and JC-5411 simultaneously for 24 h, the protein level of NLPR3, p-p65, caspase-1 p20 and IL-1β p17 were decreased when comparing with LPS treated group (Figures 3B, C). To confirm that Nrf2 was involved in the anti-inflammatory effects of JC-5411, specific Nrf2 siRNA was used. It was noticed that when *nrf2* was knockdown, the downregulating proteins including NLPR3, phosphorylated protein level of p-p65, caspase-1 p20 and IL-1β p17 effect of JC-5411 disappeared in some degree (Figures 3B, C). In addition, *in vivo* results also demonstrated that JC-5411 treatment inhibited liver NF-κB signaling through downregulation of p-p65 (Figures 3D, E). JC-5411 treatment also decreased NLRP3 and its downstream protein levels including pro-Caspase-1, Caspase-1 p10, pro-IL-1β, IL-1β p17 protein levels in the livers of ApoE^−/−^ mice (Figures 3D, E), which indicated that JC-5411 inhibited the activation of NLRP3 inflammasome *in vivo*.

Taken together, our results indicate that JC-5411 mediated anti-inflammatory effects are associated with Nrf2, NF-κB and NLRP3 inflammasome pathway both in LPS-treated J774A.1 cell and WD fed ApoE^−/−^ mice.

### JC-5411 Exerted Anti-inflammatory Effect in TNFα Induced Human Umbilical Vein Endothelial Cells via Nuclear Factor Erythroid 2-Related Factor 2 Signaling

**FIGURE 4:**
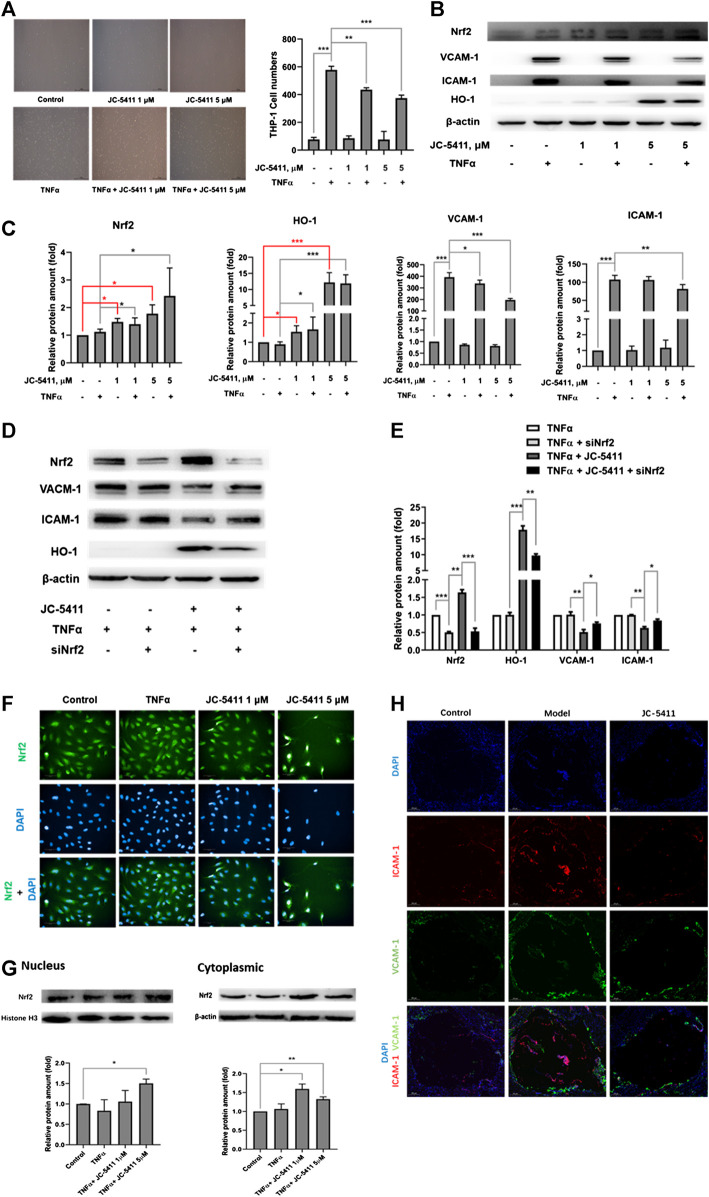
JC-5411 exerted anti-inflammatory effect in TNFα-stimulated HUVECs via Nrf2 signaling. **(A)** HUVECs were seeded in 6-well plates and pretreated with DMSO or JC-5411 (1 μM, 5 μM) for 18 h once the cells were attached to the plates. TNFα (final concentration 10 ng/ml) was then added for a further 6 h. THP-1 cells were then co-incubated with HUVECs for 30 min and the number of adhered THP-1 cells was quantified. A representative image was shown of each group in panel A, scale bars: 50 μM. Student’s *t* test were performed, **p < 0.01, ***p < 0.001, *n* = 3. **(B, C)** Western blotting was conducted to detect the protein level of Nrf2, HO-1, ICAM-1 and VCAM-1. All protein bands were normalized by *β*-actin and expressed as a fold of control. One-way ANOVA analysis was performed, *p < 0.05, **p < 0.01, ***p < 0.001, *n* = 3. **(D, E)** siNrf2 was used as described above, then cell protein of each group was collected and detected using western blotting. All protein bands were normalized by *β*-actin and expressed as a fold of control. One-way ANOVA analysis was performed, *p < 0.05, **p < 0.01, ***p < 0.001, *n* = 3. **(F)** HUVECs were treated as described above, then immunofluorescence staining was performed. A representative image was captured with High Content Analysis System. Scale bars, 50 μm; *n* = 3. **(G)** The proteins from cytoplasm and nucleus were extracted and detected by western blotting, respectively. The cytoplasm protein bands were normalized by *β*-actin, and the nucleus protein bands were normalized by histone H3, all protein bands were expressed as a fold of control, Student’s *t* test were performed, *p < 0.05, **p < 0.01, *n* = 3. **(H)** Representative immunofluorescence staining of ICAM-1 (red) and VCAM-1 (green) in aortic sinus cross sections of each treatment group where DAPI (blue) was used to highlight the cell nucleus. Scale bars, 200 μm.

Dysfunction of the endothelial cells is an important contributor to the pathobiology of atherosclerosis, and the increased secretion of adhesion molecule such as ICAM-1 and VCAM-1 in endothelial cells facilitates the macrophage adhesion to endothelial cells (Gimbrone and García-Cardeña, 2016). Monocyte adhesion assay was then performed to evaluate the anti-inflammatory effect of JC-5411 in TNFα induced HUVECs. As shown in Figure 4A, TNFα treatment remarkably increased the number of adhering THP-1 cells, while JC-5411 significantly decreased TNFα induced THP-1 cells adhesion. When *nrf2* gene was knockdown, the number of adhering THP-1 cells obviously increased compared with that of JC-5411 treated group (Supplementary Figures S3A, B), which indicated that the decreasing THP-1 adhering effect of JC-5411 was dependent on Nrf2. Meanwhile, JC-5411 increased the protein expression of Nrf2 and HO-1, but decreased the protein expression of ICAM-1 and VCAM-1 when compared with that of TNFα only induced group in HUVECs (Figures 4B, C), and this effect could be reversed when *nrf2* knockdown (Figures 4D, E).

As the translocation of Nrf2 into nucleus is a crucial part in the process of Nrf2 signaling activation (Tonelli et al., 2018), we then detected the Nrf2 expression location in HUVECs treated with JC-5411 by immunofluorescence staining assay. The results showed that JC-5411 significantly increased the Nrf2 protein level in nucleus (Figure 4F), which is in accordance with the result from cytoplasm and nucleus proteins by western blot analysis (Figure 4G).

Additionally, immunofluorescence staining showed that the expression of ICAM-1 and VCAM-1 in the aortic sinus of ApoE^−/−^ mice was significantly decreased as compared with the vehicle-treated model group (Figure 4H).

Taken together, these results indicated that JC-5411 exerts anti-inflammatory effect through activating Nrf2 signaling in HUVECs.

### JC-5411 Improved Lipid Accumulation in Liver of ApoE^−/−^ Mice

**FIGURE 5:**
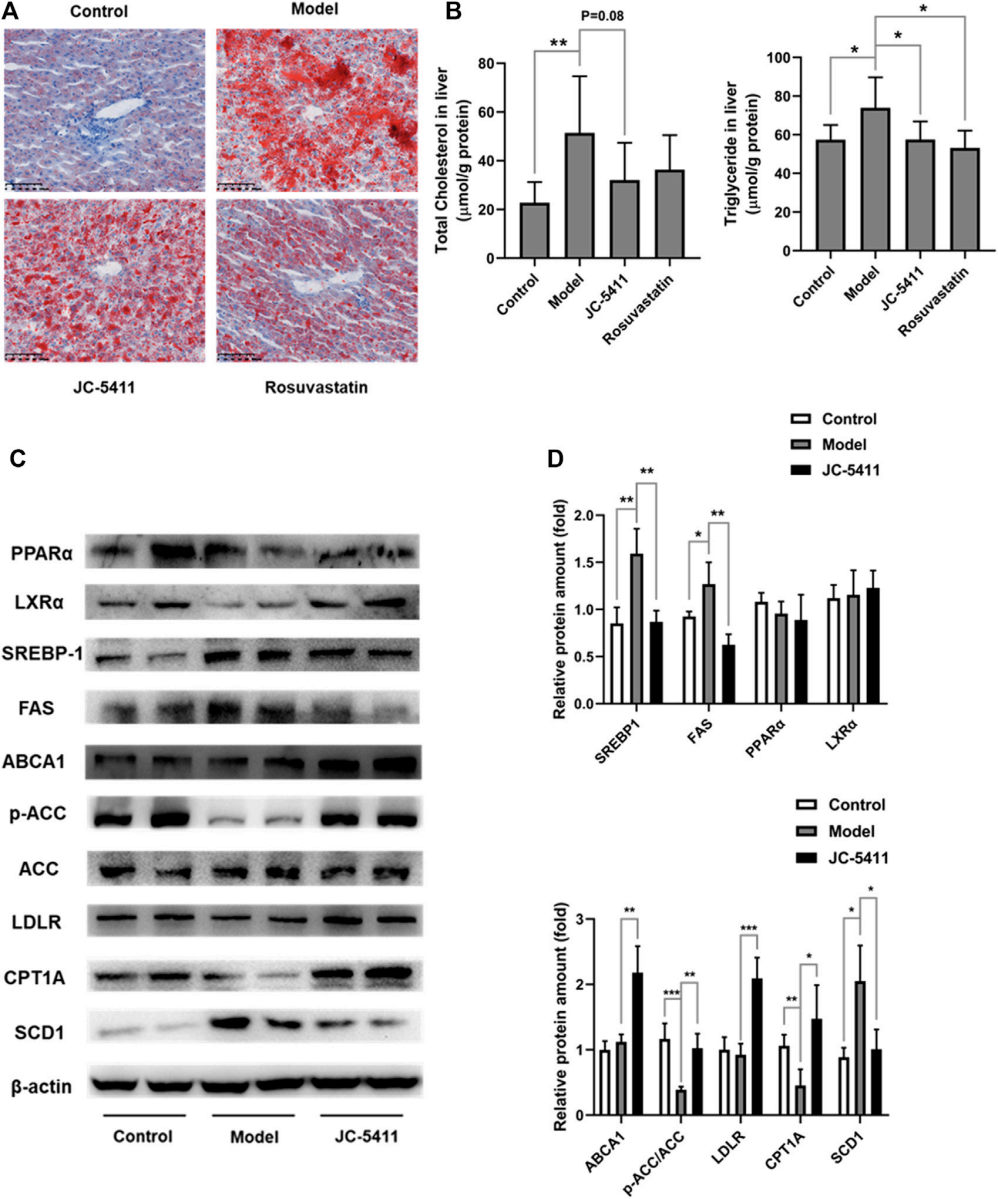
JC-5411 treatment improved lipid metabolism in ApoE^−/−^ mice. **(A)** Representative images of liver sections of ApoE^−/−^ mice stained by ORO. Scale bars, 100 μm. **(B)** The levels of TC and TG in the livers of ApoE^−/−^ mice. One-way ANOVA was performed, *p < 0.05, **p < 0.01, *n* = 6–8 per group. **(C, D)** The protein expression levels of PPARα, LXRα, SREBP-1, ABCA1, p-ACC, ACC, LDLR, CPT1A and SCD1 were detected by western blot, respectively. Representative images were shown. All protein bands were normalized by *β*-actin and expressed as a fold of control. Student’s *t* test, *p < 0.05, **p < 0.01, ***p < 0.001, *n* = 4 per group.

Abnormal lipid accumulation also plays a key role in the pathology of atherosclerosis (Moore et al., 2013), and lipid accumulation is closely linked to inflammation. (de Jager and Pasterkamp, 2013). As mentioned above, JC-5411 decreased serum VLDL and TG levels in serum of ApoE^−/−^ mice (Figures 2B, C). Therefore, we examined whether treatment with JC-5411 could restore lipid metabolism in liver of ApoE^−/−^ mice. As shown in Figure 5A, the ORO staining of ApoE^−/−^ livers from JC-5411 treatment mice showed significantly less positive area than that of the model group. In addition, JC-5411 treatment decreased TG (*p* < 0.05) and TC (*p* = 0.08) levels in liver in comparison with the model group in ApoE^−/−^ mice (Figure 5B).

To further investigate this improvement of lipid metabolism by JC-5411 treatment in ApoE^−/−^ mice, we decided to evaluate the liver protein expression levels of lipid metabolism related genes. ATP-binding cassette transporter A1 (ABCA1) and low-density lipoprotein receptor (LDLR) are two crucial proteins within the process of cholesterol transfer (Yu et al., 2019). The results showed that JC-5411 treatment significantly increased the protein expression levels of ABCA1 and LDLR in the livers of ApoE^−/−^ mice when compared with model group (Figures 5C, D). Moreover, JC-5411 treatment decreased the protein level of SCD1 (an enzyme that catalyzes the synthesis of fatty acids (ALJohani et al., 2017)), but increased the inactivation form of the rate-limiting enzyme of *de novo* fatty acid synthesis p-ACC (Kim et al., 2017) and the fatty acid β-oxidation CPT1A (Pavethynath et al., 2019) protein levels when compared with model group (Figures 5C, D). What’s more, JC-5411 treatment also decreased the protein level of fatty acid synthase (FAS), one of the important lipogenic genes (Wang and Tontonoz, 2018), but did not affect the protein level of sterol regulatory element-binding protein 1 (SREBP-1), peroxisome proliferator-activated receptor α (PPARα) and Liver X receptors α (LXRα) (Figures 5C, D). These observations indicate that JC-5411 is capable hindering lipid accumulation and accelerates fatty acid β-oxidation in liver of ApoE^−/−^ mice.

### JC-5411 Improved Lipid Metabolism in Hyperlipidemic Golden Hamsters

Hamster has been found a better animal model in lipid metabolism study because of its more similar feature of lipid biological process to human (Jiang et al., 2013). Therefore, the regulating effects of lipid metabolism by JC-5411 were further evaluated in hyperlipidemic golden hamsters. The serum levels of TG, TC and LDL-C from all three dose groups of JC-5411 were significantly lower than that of the model group (Supplementary Figure S3A). There was no significant change in serum HDL-C levels between JC-5411 20 mg/kg, 40 mg/kg treatment groups and the model group while in the 60 mg/kg dose group which showed a lower level than in model group (Supplementary Figure S3A). The ORO staining result of JC-5411 in golden hamster livers also showed significantly fewer positive areas as compared with the model group (Supplementary Figure S3B). In addition, JC-5411 significantly decreased the levels of TG and TC in the liver as compared with the model group in hyperlipidemic golden hamsters (Supplementary Figure S3C). JC-5411 improved the steatosis of liver cells compared with model group by H&E staining (Supplementary Figure S3D), which is identical with the result observed in ApoE^−/−^ mice. These results demonstrate that JC-5411 treatments reduce the lipid accumulation in dyslipidemic golden hamsters.

### JC-5411 Increased the Antioxidant Capacity in ApoE^−/−^ Mice and in H_2_O_2_ Stimulated Human Umbilical Vein Endothelial Cells

Nrf2 plays an important role in protecting cells from the damage caused by oxidative stress (Jakobs et al., 2017). Oxidative stress is another factor involved in the pathogenesis of atherosclerosis. What’s more, inflammation and oxidative stress help each other forward when causing tissue damage in atherosclerosis, and abnormal lipid accumulate aggravates inflammatory and oxidative stress state *in vivo* (Olechnowicz et al., 2018). We thus investigated the protective characteristics of JC-5411 using a DHE assay to determine the superoxide (O_2_^−^) formation in atherosclerotic plaques of ApoE^−/−^ mice. As expected, JC-5411 could decrease the positive area ratio (fluorescence area in plaque/total area of plaque) in comparison with model group (Supplementary Figures S4A, B), suggesting that JC-5411 is able to reduce superoxide levels in atherosclerotic plaques. In parallel, JC-5411 treatment increased the SOD activity and decreased the MDA level in the livers of ApoE^−/−^ mice (Supplementary Figures 4C, D). Additionally, JC-5411 treatment significantly increased the antioxidant genes’ mRNA levels of Nrf2 downstream including NAD(P)H quinone dehydrogenase 1 (NQO1), glutamate synthesis related genes, glutamate-cysteine ligase catalytic subunit (GCLC) and glutamate-cysteine ligase modifier subunit (GCLM) (Chapple et al., 2012) in liver of ApoE^−/−^ mice (Supplementary Figure S4E).

Then HUVECs stimulated with H_2_O_2_ was used to establish the oxidative stress cell model. As shown in Figures 4F–I, JC-5411 increased the mRNA level of antioxidant related genes including Nrf2, NQO1, GCLC and GCLM when compared with H_2_O_2_ group. What’s more, these genes decreased significantly in nrf2 knockdown group when compared with JC-5411-treated group, which means JC-5411 also increased the antioxidant capacity in H_2_O_2_-treated HUVECs via Nrf2 signaling. Our results indicate that JC-5411 treatment activates Nrf2 signaling and increases the antioxidant capacity in WD fed ApoE^−/−^ mice and H_2_O_2_ stimulated HUVECs.

## Discussion

**FIGURE 6:**
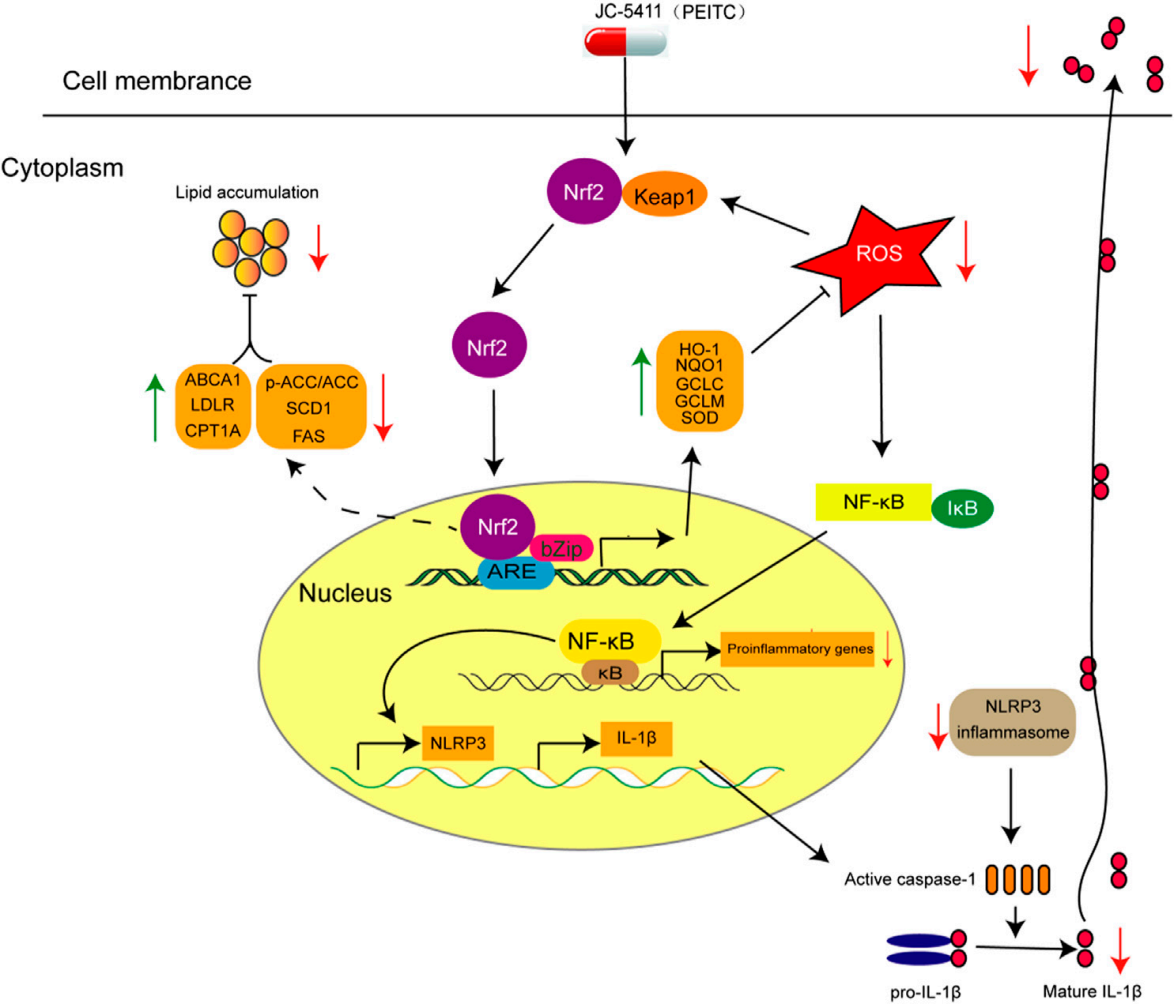
The proposed mechanism of action of JC-5411 via activation of Nrf2 pathway against arteriosclerosis. Specifically, JC-5411 exerts anti-inflammatory effect by inhibiting the activation of NF-κB and NLRP3 inflammasome signaling, thus reducing the release of IL-1β, and its anti-inflammatory effect was dependent on activating Nrf2. In addition, JC-5411 improves lipid metabolism by increasing the expression level of cholesterol transfer genes (ABCA1 and LDLR), decreasing the fatty acid synthesis genes (SCD1 and FAS), and increasing inactivation form of the rate-limiting enzyme of *de novo* fatty acid synthesis (p-ACC) and fatty acid β-oxidation genes (CPT1A) in ApoE^−/−^ mice. Moreover, JC-5411 has antioxidant capacity by activating Nrf2 pathway and thus increasing the expression of various antioxidant genes including HO-1, NQO1, GCLC and GCLM in both ApoE^−/−^ mice and HUVECs. Taken together, JC-5411 as a Nrf2 activator protects against atherosclerosis through antiinflammation, regulating lipid metabolism and antioxidation.

Atherosclerosis is a chronic disease characterized with the formation of atheromatous plaques in arteries (Libby et al., 2011). Several complex pathogenetic processes are involved in the development and progression of the disease, which include inflammation, lipid metabolism disorder and oxidative stress (Libby et al., 2011; Forstermann et al., 2017). In this study, JC-5411 can increase the expression of Nrf2 and its downstream gene HO-1 *in vitro* and in the liver of ApoE^−/−^ mice, suggesting that JC-5411 upregulates Nrf2 signaling. We then showed, for the first time, that JC-5411 as a Nrf2 activator was able to effectively attenuate atherosclerotic lesions in ApoE^−/−^ mouse model fed on WD. We found that this reduction is achieved via attenuating inflammatory activities, rebalancing lipid metabolism and increasing the capacity of antioxidation (Figure 6). Importantly, we provide the first direct evidence that JC-5411 has the potential to treat hyperlipidemia observed through animal models including WD induced ApoE^−/−^ mice and hyperlipidemic golden hamsters.

It has been demonstrated that inflammation facilitates the pathogenetic process of lesion formation in atherosclerosis (Libby, 2012) in which Nrf2 signaling plays a critical role (Mimura and Itoh, 2015; Zakkar et al., 2009; Boyanapalli et al., 2014). In this study, we found that JC-5411 was effective against inflammation in ApoE^−/−^ mouse model fed with WD, LPS induced macrophage J774A.1 cell and TNFα stimulated HUVECs. We demonstrated that JC-5411 could attenuate monocyte adhesion in TNFα induced HUVECs and decrease inflammatory factors’ secretion in LPS stimulated macrophages via Nrf2 dependent manner (Figures 3, 4). We then demonstrated that JC-5411 positively affected Nrf2 signaling through inhibiting the activation of NF-κB and NLRP3 inflammasome pathway both in ApoE^−/−^ mouse and LPS induced macrophage J774A.1 cell. Since NF-κB signaling plays an important role in the primary stage of NLRP3 inflammasome activation which in turn is suppressed by the activation of Nrf2 signaling (Grebe et al., 2018; Dayalan Naidu et al., 2018; Ahmed et al., 2017; Juurlink, 2012), we concluded that the anti-inflammatory activities of JC-5411 in WD fed ApoE^−/−^ mice and *in vitro* were via its regulation on the Nrf2/NF-κB/NLRP3 inflammasome signaling.

Dysregulation of lipid metabolism is the central part in the progression of atherosclerosis (Libby et al., 2011; Lu and Daugherty, 2015). In animal study, we chosen rosuvastatin as positive control. Both JC-5411 and rosuvastatin could decrease the serum TC level in ApoE^−/−^ mice (Figure 5A), but our data showed that JC-5411 had different regulating lipid metabolism mechanism compared with rosuvastatin. As reported, rosuvastatin exerts that effect via competitively inhibiting the 3-hydroxy-3-methylglutaryl coenzyme A reductase, a liver enzyme responsible of the rate-limiting step in cholesterol synthesis, and this effect contributes a lot in attenuating atherosclerosis development (Cortese et al., 2016). While the effect of JC-5411 on lipid metabolism was through increasing cholesterol transfer gene (ABCA1 and LDLR) expression and decreasing fatty acids synthesis related gene (SCD1 and FAS) expression, and increasing the inactivation form of rate-limiting enzyme of *de novo* fatty acid synthesis (p-ACC) and fatty acids β-oxidation (CPT1A) in the liver of ApoE^−/−^ mice. Though many studies have suggested the association of lipid metabolism with Nrf2 signaling (Mimura and Itoh, 2015; Tanaka et al., 2008), we failed to demonstrate the direct relationship between JC-5411 and these lipid metabolism related genes such as ABCA1 and LDLR when we knocked down *nrf2* in JC-5411 treated HepG2 cells with *nrf2* specific siRNA (date not shown). Further investigation is required to elucidate how JC-5411 mediated lipid metabolism is involved in Nrf2 signaling.

As liver plays a pivotal role in lipid metabolism (You and Arteel, 2019), a high level of lipid within the liver facilitates the formation of lipotoxic species and induces the release of chemokines and pre-inflammatory cytokines such as TNFα, IL-1β and IL-6. Furthermore, it is now accepted that these lipotoxic species also contribute to oxidative stress damage in liver cells (Friedman et al., 2018; Byrne and Targher, 2015; Marra and Svegliati-Baroni, 2018; Buzzetti et al., 2016). Our data showed that JC-5411 could restore the balance of lipid metabolism in WD-fed ApoE^−/−^ mice, which would be beneficial to decrease oxidative stress and attenuate inflammation. Therefore, it is concluded that JC-5411 exerts the anti-inflammatory and antioxidant effects within liver of WD-fed ApoE^−/−^ mice may result from the indirect rebalancing of the lipid accumulation in liver of JC-5411.

In addition, considering oxidative stress can be regulated by Nrf2 signaling, we assume that the increased activity of SOD and decreased expression of MDA in the liver by JC-5411 could be, at least in part, through upregulation of Nrf2 signaling, which is in line with the results of previous reports by other Nrf2 activators (Du et al., 2016; Wu et al., 2017; Wang et al., 2018). Since the production of O_2_^−^, which can be regulated by Nrf2 signaling (Ashino et al., 2013), is strongly associated with the pathological process of atherosclerosis (Vendrov et al., 2007; Lazaro et al., 2018), reduction in the accumulation or concentration O_2_^−^ in atherosclerotic plaques by JC-5411 gives further evidence that this compound has the potential to become an important therapy for atherosclerosis.

In summary, we have shown that JC-5411 can act as a Nrf2 activator which in turn inhibits the progression of atherosclerosis in ApoE^−/−^ mice, and demonstrate that this inhibition is achieved through multiple mechanisms including the attenuation of inflammation, regulation of dysregulated lipid metabolism and increase in antioxidant activity. Although the role that Nrf2 plays in atherosclerosis is still disputable (Mimura and Itoh, 2015; Freigang et al., 2011; Ruotsalainen et al., 2013), some Nrf2 activators have been shown to exhibit marked activity in combatting atherosclerosis (Zhu et al., 2019; Aboonabi and Singh, 2015; Xie et al., 2016; Lazaro et al., 2018). Even so, our findings provide further evidence that JC-5411, as a Nrf2 activator, might have the potential to be an effective therapy for atherosclerosis and its related cardiovascular diseases.

## Data Availability Statement

All datasets presented in this study are included in the article/Supplementary Material.

## Author Contributions

SS, JC, and Yanni X contributed to the conception of the study. XJ contributed significantly to perform the experiments, analysis and manuscript preparation. YL, WW, XH, MC, JH and JZ helped perform the experiments. CW, SL, JL, XW, and YX helped perform the analysis with constructive discussions.

## Funding

This work was supported by the grants from CAMS Fundamental Research Funds (2019-RC-HL-009), National Natural Science Foundation of China (81573482 and 81973328), Beijing-Tianjin-Hebei Basic Research Cooperation Project (H2019205318), CAMS Innovation Fund for Medical Sciences (2016-I2M-1-011), and the Drug Innovation Major Project (2018ZX09711001-003-006, 2018ZX09735001-002-001 and 2018ZX09711001-007-002).

## Conflict of Interest

Author JC was employed by company ^2^JC (Wuxi) COMPANY, Inc.

The remaining authors declare that the research was conducted in the absence of any commercial or financial relationships that could be construed as a potential conflict of interest.

## References

[B1] Aboonabi A. Singh I. (2015). Chemopreventive role of anthocyanins in atherosclerosis via activation of Nrf2-ARE as an indicator and modulator of redox *.* Biomed. Pharmacother. 72, 30–36. 10.1016/j.biopha.2015.03.008 26054672

[B2] Ahmed S. M. U. Luo L. Namani A. Wang X. J. Tang X. (2017). Nrf2 signaling pathway: pivotal roles in inflammation *.* Biochim Biophys. Acta. Mol. Basis. Dis. 1863 (2), 585–597. 10.1016/j.bbadis.2016.11.005 27825853

[B3] ALJohani A. M. Syed D. N. Ntambi J. M. (2017). Insights into stearoyl-CoA desaturase-1 regulation of systemic metabolism *.* Trends Endocrinol. Metab. 28 (12), 831–842. 10.1016/j.tem.2017.10.003 29089222 PMC5701860

[B4] Ashino T. Yamamoto M. Yoshida T. Numazawa S. (2013). Redox-sensitive transcription factor Nrf2 regulates vascular smooth muscle cell migration and neointimal hyperplasia *.* Arterioscler. Thromb. Vasc. Biol. 33 (4), 760–768. 10.1161/atvbaha.112.300614 23413426

[B5] Bäck M. Yurdagul A. Tabas I. Öörni K. Kovanen P. T. (2019). Inflammation and its resolution in atherosclerosis: mediators and therapeutic opportunities *.* Nat. Rev. Cardiol. 16 (7), 389–406. 10.1038/s41569-019-0169-2 30846875 PMC6727648

[B6] Baldrighi M. Mallat Z. Li X. (2017). NLRP3 inflammasome pathways in atherosclerosis *.* Atherosclerosis 267, 127–138. 10.1016/j.atherosclerosis.2017.10.027 29126031

[B7] Bauernfeind F. G. Horvath G. Stutz A. Alnemri E. S. MacDonald K. (2009). Cutting edge: NF-kappaB activating pattern recognition and cytokine receptors license NLRP3 inflammasome activation by regulating NLRP3 expression *.* J. Immunol. 183 (2), 787–791. 10.4049/jimmunol.0901363 19570822 PMC2824855

[B8] Boyanapalli S. S. S. Paredes-Gonzalez X. Fuentes F. Zhang C. Guo Y. Pung D. (2014). Nrf2 knockout attenuates the anti-inflammatory effects of phenethyl isothiocyanate and curcumin *.* Chem. Res. Toxicol. 27 (12), 2036–2043. 10.1021/tx500234h 25387343 PMC4269407

[B9] Buzzetti E. Pinzani M. Tsochatzis E. A. (2016). The multiple-hit pathogenesis of non-alcoholic fatty liver disease (NAFLD) *.* Metabolism 65 (8), 1038–1048. 10.1016/j.metabol.2015.12.012 26823198

[B10] Byrne C. D. Targher G. (2015). NAFLD: a multisystem disease *.* J. Hepatol. 62 (Suppl. 1), S47–S64. 10.1016/j.jhep.2014.12.012 25920090

[B11] Chapple S. J. Siow R. C. M. Mann G. E. (2012). Crosstalk between Nrf2 and the proteasome: therapeutic potential of Nrf2 inducers in vascular disease and aging *.* Int. J. Biochem. Cell Biol. 44 (8), 1315–1320. 10.1016/j.biocel.2012.04.021 22575091

[B12] Chen N. Frishman W. H. (2016). High-density lipoprotein infusion therapy and atherosclerosis: current Research and future directions *.* Cardiol. Rev. 24 (6), 298–302. 10.1097/crd.0000000000000111 27465534

[B13] Cortese F. Gesualdo M. Cortese A. Carbonara S. Devito F. Zito A. (2016). Rosuvastatin: beyond the cholesterol-lowering effect *.* Pharmacol. Res. 107, 1–18. 10.1016/j.phrs.2016.02.012 26930419

[B14] Cuadrado A. Rojo A. I. Wells G. Hayes J. D. Cousin S. P. Rumsey W. L. (2019). Therapeutic targeting of the NRF2 and KEAP1 partnership in chronic diseases *.* Nat. Rev. Drug Discov. 18 (4), 295–317. 10.1038/s41573-018-0008-x 30610225

[B15] Dayalan Naidu S. Suzuki T. Yamamoto M. Fahey J. W. Dinkova-Kostova A. T. (2018). Phenethyl isothiocyanate, a dual activator of transcription factors NRF2 and HSF1 *.* Mol. Nutr. Food Res. 62 (18), e1700908 10.1002/mnfr.201700908 29710398 PMC6175120

[B16] de Jager S. C. A. Pasterkamp G. (2013). Crosstalk of lipids and inflammation in atherosclerosis: the PRO of PGRN? Cardiovasc. Res. 100 (1), 4–6. 10.1093/cvr/cvt199 23946497

[B17] Du J. Zhang M. Lu J. Zhang X. Xiong Q. Xu Y. (2016). Osteocalcin improves nonalcoholic fatty liver disease in mice through activation of Nrf2 and inhibition of JNK *.* Endocrine 53 (3), 701–709. 10.1007/s12020-016-0926-5 26994931

[B18] Förstermann U. Xia N. Li H. (2017) Roles of vascular oxidative stress and nitric oxide in the pathogenesis of atherosclerosis *.* Circ. Res. 120 (4), 713–735. 10.1161/circresaha.116.309326 28209797

[B19] Freigang S. Ampenberger F. Spohn G. Heer S. Shamshiev A. T. Kisielow J. (2011). Nrf2 is essential for cholesterol crystal-induced inflammasome activation and exacerbation of atherosclerosis *.* Eur. J. Immunol. 41 (7), 2040–2051. 10.1002/eji.201041316 21484785

[B20] Friedman S. L. Neuschwander-Tetri B. A. Rinella M. Sanyal A. J. (2018). Mechanisms of NAFLD development and therapeutic strategies *.* Nat. Med. 24 (7), 908–922. 10.1038/s41591-018-0104-9 29967350 PMC6553468

[B21] Gimbrone M. A. Jr. García-Cardeña G. (2016). Endothelial cell dysfunction and the pathobiology of atherosclerosis *.* Circ. Res. 118 (4). 620–636. 10.1161/circresaha.115.306301 26892962 PMC4762052

[B22] Grebe A. Hoss F. Latz E. (2018). NLRP3 inflammasome and the IL-1 pathway in atherosclerosis *.* Circ. Res. 122 (12), 1722–1740. 10.1161/circresaha.118.311362 29880500

[B23] Hansson G. K. Robertson A.-K. L. Söderberg-Nauclér C. (2006). Inflammation and atherosclerosis *.* Annu. Rev. Pathol. 1, 297–329. 10.1146/annurev.pathol.1.110304.100100 18039117

[B24] Hoseini Z. Sepahvand F. Rashidi B. Sahebkar A. Masoudifar A. Mirzaei H. (2018). NLRP3 inflammasome: its regulation and involvement in atherosclerosis *.* J. Cell. Physiol. 233 (3), 2116–2132. 10.1002/jcp.25930 28345767

[B25] Huang C.-S. Lin A.-H. Liu C.-T. Tsai C.-W. Chang I.-S. Chen H.-W. (2013). Isothiocyanates protect against oxidized LDL-induced endothelial dysfunction by upregulating Nrf2-dependent antioxidation and suppressing NFkappaB activation *.* Mol. Nutr. Food Res. 57 (11), 1918–1930. 10.1002/mnfr.201300063 23836589

[B26] Jakobs P. Serbulea V. Leitinger N. Eckers A. Haendeler J. (2017). Nuclear factor (Erythroid-Derived 2)-like 2 and thioredoxin-1 in atherosclerosis and ischemia/reperfusion injury in the heart *.* Antioxidants Redox Signal. 26 (12), 630–644. 10.1089/ars.2016.6795 PMC5397216 27923281

[B27] Ji Y. Kuo Y. Morris M. E. (2005). Pharmacokinetics of dietary phenethyl isothiocyanate in rats *.* Pharm. Res. (N. Y.) 22 (10), 1658–1666. 10.1007/s11095-005-7097-z 16180123

[B28] Jiang C. Y. Yang K.-M. Yang L. Miao Z.-X. Wang Y.-H. Zhu H.-B. (2013). A (1)H NMR-based metabonomic investigation of time-related metabolic trajectories of the plasma, urine and liver extracts of hyperlipidemic hamsters *.* PLoS One 8 (6), e66786 10.1371/journal.pone.0066786 23840531 PMC3694122

[B29] Juurlink B. H. J. (2012). Dietary Nrf2 activators inhibit atherogenic processes *.* Atherosclerosis 225 (1), 29–33. 10.1016/j.atherosclerosis.2012.08.032 22986182

[B30] Kattoor A. J. Pothineni N. V. K. Palagiri D. Mehta J. L. (2017). Oxidative stress in atherosclerosis *.* Curr. Atheroscler. Rep. 19 (11), 42 10.1007/s11883-017-0678-6 28921056

[B31] Kim C.-W. Addy C. Kusunoki J. Anderson N. N. Deja S. Fu X. (2017). Acetyl CoA carboxylase inhibition reduces hepatic steatosis but elevates plasma triglycerides in mice and humans: a bedside to bench investigation *.* Cell Metab. 26 (2), 394–406. 10.1016/j.cmet.2017.07.009 28768177 PMC5603267

[B32] Lazaro I. Lopez-Sanz L. Bernal S. Oguiza A. Recio C. Melgar A. (2018). Nrf2 activation provides atheroprotection in diabetic mice through concerted upregulation of antioxidant, anti-inflammatory, and autophagy mechanisms *.* Front. Pharmacol. 9, 819 10.3389/fphar.2018.00819 30108504 PMC6080546

[B33] Libby P. (2012). Inflammation in atherosclerosis *.* Arterioscler. Thromb. Vasc. Biol. 32 (9), 2045–2051. 10.1161/atvbaha.108.179705 22895665 PMC3422754

[B34] Libby P. Bornfeldt K. E. Tall A. R. (2016). Atherosclerosis: successes, surprises, and future challenges *.* Circ. Res. 118 (4), 531–534. 10.1161/circresaha.116.308334 26892955 PMC4762065

[B35] Libby P. Ridker P. M. Hansson G. K. (2011). Progress and challenges in translating the biology of atherosclerosis *.* Nature 473 (7347), 317–325. 10.1038/nature10146 21593864

[B36] Lu H. Daugherty A. (2015). Atherosclerosis. Arterioscler. Thromb. Vasc Biol 35 (3), 485–491. 10.1161/atvbaha.115.305380 25717174 PMC4511379

[B37] Marra F. Svegliati-Baroni G. (2018). Lipotoxicity and the gut-liver axis in NASH pathogenesis *.* J. Hepatol. 68 (2), 280–295. 10.1016/j.jhep.2017.11.014 29154964

[B38] Mimura J. Itoh K. (2015). Role of Nrf2 in the pathogenesis of atherosclerosis *.* Free Radic. Biol. Med. 88 (Pt B), 221–232. 10.1016/j.freeradbiomed.2015.06.019 26117321

[B39] Moore K. J. Sheedy F. J. Fisher E. A. (2013). Macrophages in atherosclerosis: a dynamic balance *.* Nat. Rev. Immunol. 13 (10), 709–721. 10.1038/nri3520 23995626 PMC4357520

[B40] Olechnowicz J. Tinkov A. Skalny A. Suliburska J. (2018). Zinc status is associated with inflammation, oxidative stress, lipid, and glucose metabolism *.* J. Physiol. Sci. 68 (1), 19–31. 10.1007/s12576-017-0571-7 28965330 PMC5754376

[B41] Pavethynath S. Imai C. Jin X. Hichiwa N. Takimoto H. Okamitsu M. (2019). Metabolic and immunological shifts during mid-to-late gestation influence maternal blood methylation of CPT1A and SREBF1 *.* Int. J. Mol. Sci. 20 (5), 1066 10.3390/ijms20051066 PMC6429071 30823689

[B42] Próchnicki T. Latz E. (2017). Inflammasomes on the crossroads of innate immune recognition and metabolic control *.* Cell Metabol. 26 (1), 71–93. 10.1016/j.cmet.2017.06.018 28683296

[B43] Ruotsalainen A.-K. Inkala M. Partanen M. E. Lappalainen J. P. Kansanen E. Mäkinen P. I. (2013). The absence of macrophage Nrf2 promotes early atherogenesis *.* Cardiovasc. Res. 98 (1), 107–115. 10.1093/cvr/cvt008 23341579

[B44] Tanaka Y. Aleksunes L. M. Yeager R. L. Gyamfi M. A. Esterly N. Guo G. L. (2008), NF-E2-related factor 2 inhibits lipid accumulation and oxidative stress in mice fed a high-fat diet *.* J. Pharmacol. Exp. Ther. 325 (2), 655–664. 10.1124/jpet.107.135822 18281592

[B45] Tonelli C. Chio I. I. C. Tuveson D. A. (2018). Transcriptional regulation by Nrf2 *.* Antioxidants Redox Signal. 29 (17), 1727–1745. 10.1089/ars.2017.7342 PMC6208165 28899199

[B46] Vendrov A. E. Hakim Z. S. Madamanchi N. R. Rojas M. Madamanchi C. Runge M. S. (2007). Atherosclerosis is attenuated by limiting superoxide generation in both macrophages and vessel wall cells *.* Arterioscler. Thromb. Vasc. Biol. 27 (12), 2714–2721. 10.1161/atvbaha.107.152629 17823367

[B47] Wang B. Tontonoz P. (2018). Liver X receptors in lipid signalling and membrane homeostasis *.* Nat. Rev. Endocrinol. 14 (8), 452–463. 10.1038/s41574-018-0037-x 29904174 PMC6433546

[B48] Wang W. Zhang Y. Li H. Zhao Y. Cai E. Zhu H. (2018). Protective effects of sesquiterpenoids from the root of panax ginseng on fulminant liver injury induced by lipopolysaccharide/d-galactosamine *.* J. Agric. Food Chem. 66 (29), 7758–7763. 10.1021/acs.jafc.8b02627 29974747

[B49] Wolf D. Ley K. (2019). Immunity and inflammation in atherosclerosis *.* Circ. Res. 124 (2), 315–327. 10.1161/circresaha.118.313591 30653442 PMC6342482

[B50] Wu M. Y. Li C.-J. Hou M.-F. Chu P.-Y. (2017a). New insights into the role of inflammation in the pathogenesis of atherosclerosis *.* Int. J. Mol. Sci. 18 (10). 10.3390/ijms18102034 PMC5666716 28937652

[B51] Wu T. Li J. Li Y. Song H. (2017b). Antioxidant and hepatoprotective effect of swertiamarin on carbon tetrachloride-induced hepatotoxicity via the Nrf2/HO-1 pathway *.* Cell. Physiol. Biochem. 41 (6), 2242–2254. 10.1159/000475639 28448964

[B52] Xie L. Gu Y. Wen M. Zhao S. Wang W. Ma Y. (2016). Hydrogen sulfide induces Keap1 S-sulfhydration and suppresses diabetes-accelerated atherosclerosis via Nrf2 activation *.* Diabetes 65 (10), 3171–3184. 10.2337/db16-0020 27335232 PMC8928786

[B53] Xu Y. Liu P. Xu S. Koroleva M. Zhang S. Si S. (2017). Tannic acid as a plant-derived polyphenol exerts vasoprotection via enhancing KLF2 expression in endothelial cells *.* Sci. Rep. 7 (1), 6686 10.1038/s41598-017-06803-x 28751752 PMC5532219

[B54] Yamamoto M. Kensler T. W. Motohashi H. (2018). The KEAP1-NRF2 system: a thiol-based sensor-effector apparatus for maintaining redox homeostasis *.* Physiol. Rev. 98 (3), 1169–1203. 10.1152/physrev.00023.2017 29717933 PMC9762786

[B55] You M. Arteel G. E. (2019). Effect of ethanol on lipid metabolism *.* J. Hepatol. 70 (2), 237–248. 10.1016/j.jhep.2018.10.037 30658725 PMC6436537

[B56] Yu X.-H. Zhang D.-W. Zheng X.-L. Tang C.-K. (2019). Cholesterol transport system: an integrated cholesterol transport model involved in atherosclerosis *.* Prog. Lipid Res. 73, 65–91. 10.1016/j.plipres.2018.12.002 30528667

[B57] Zakkar M. Van der Heiden K. Luong L. A. Chaudhury H. Cuhlmann S. Hamdulay S. S. (2009). Activation of Nrf2 in endothelial cells protects arteries from exhibiting a proinflammatory state *.* Arterioscler. Thromb. Vasc. Biol. 29 (11), 1851–1857. 10.1161/atvbaha.109.193375 19729611

[B58] Zhang L. Zhang H. Li X. Jia B. Yang Y. Zhou P. (2016). Miltirone protects human EA.hy926 endothelial cells from oxidized low-density lipoprotein-derived oxidative stress via a heme oxygenase-1 and MAPK/Nrf2 dependent pathway *.* Phytomedicine 23 (14), 1806–1813. 10.1016/j.phymed.2016.11.003 27912883

[B59] Zhu Y. Zhang Y. Huang X. Xie Y. Qu Y. Long H. (2019). Z-Ligustilide protects vascular endothelial cells from oxidative stress and rescues high fat diet-induced atherosclerosis by activating multiple NRF2 downstream genes *.* Atherosclerosis 284, 110–120. 10.1016/j.atherosclerosis.2019.02.010 30897380

