# Supplementary figures and images for: Nuclear Factor Erythroid 2 Related Factor 2 Activator JC-5411 Inhibits Atherosclerosis Through Suppression of Inflammation and Regulation of Lipid Metabolism
Source: Front Pharmacol. 2020 Nov 16;11:532568. doi: 10.3389/fphar.2020.532568 (PMC7797784; doi:10.3389/fphar.2020.532568)

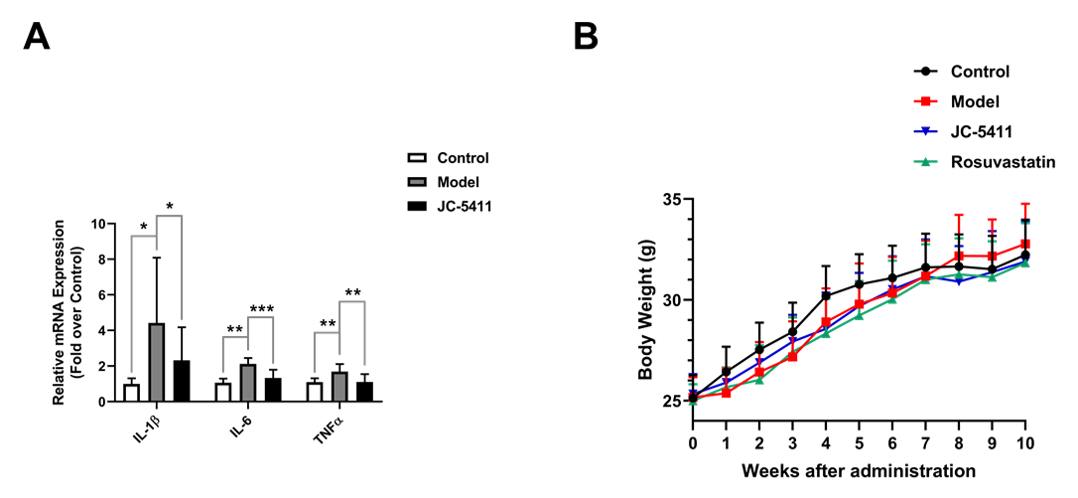

Supplement: Supplementary file 1 [file image1.tif]

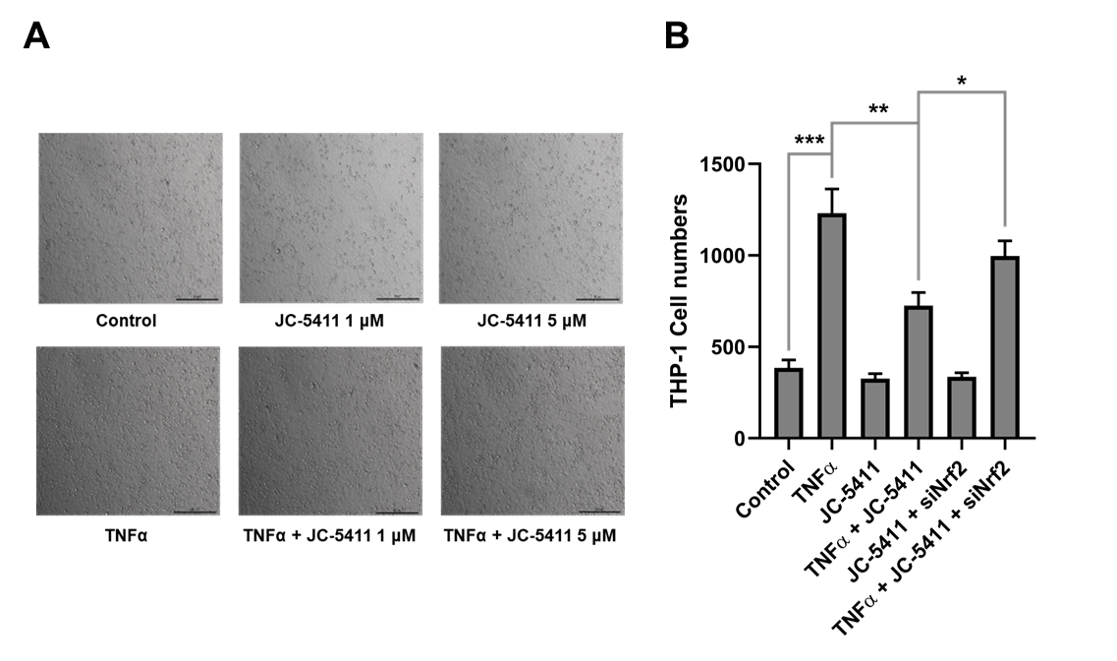

Supplement: Supplementary file 2 [file image2.tif]

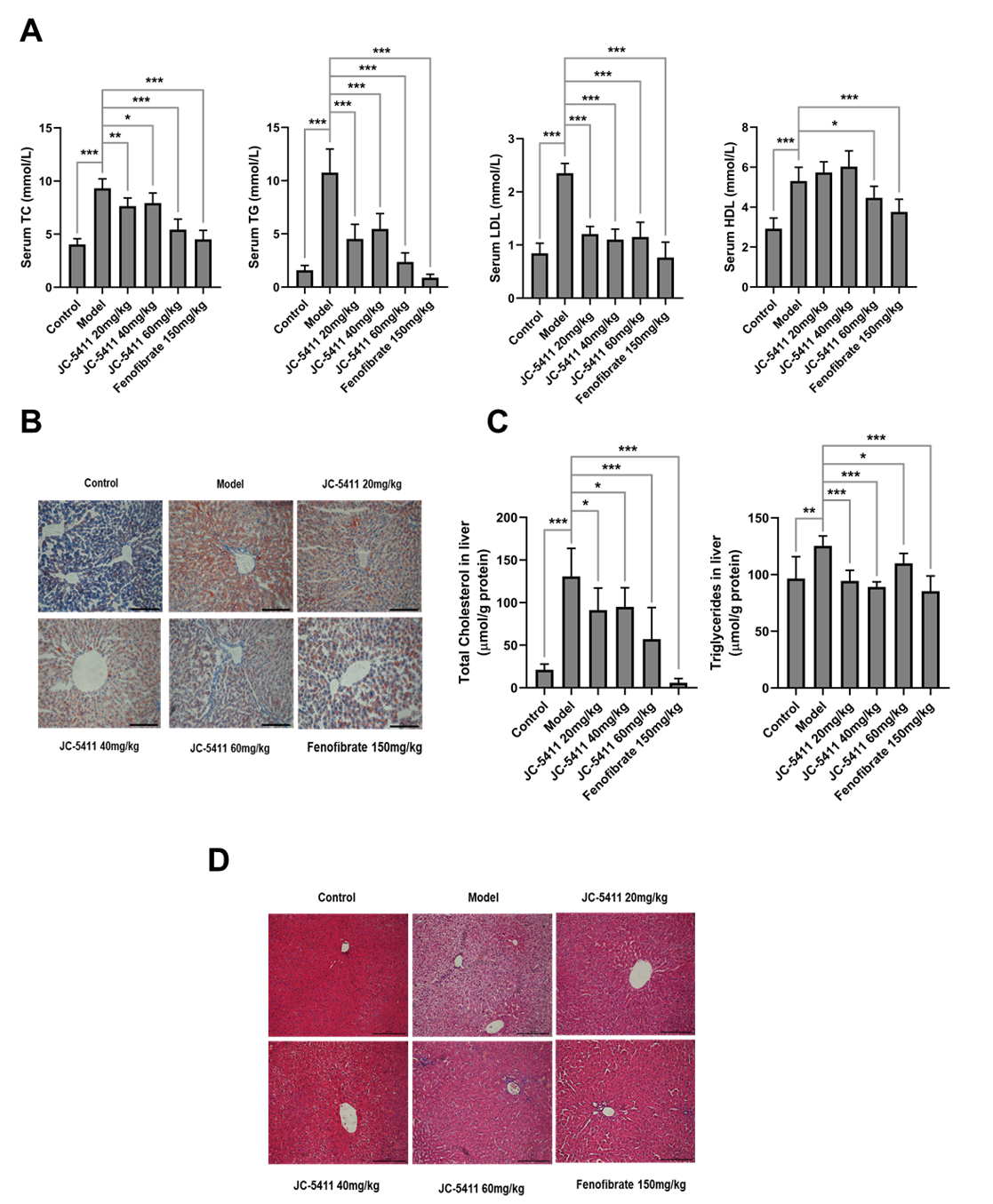

Supplement: Supplementary file 3 [file image3.tif]

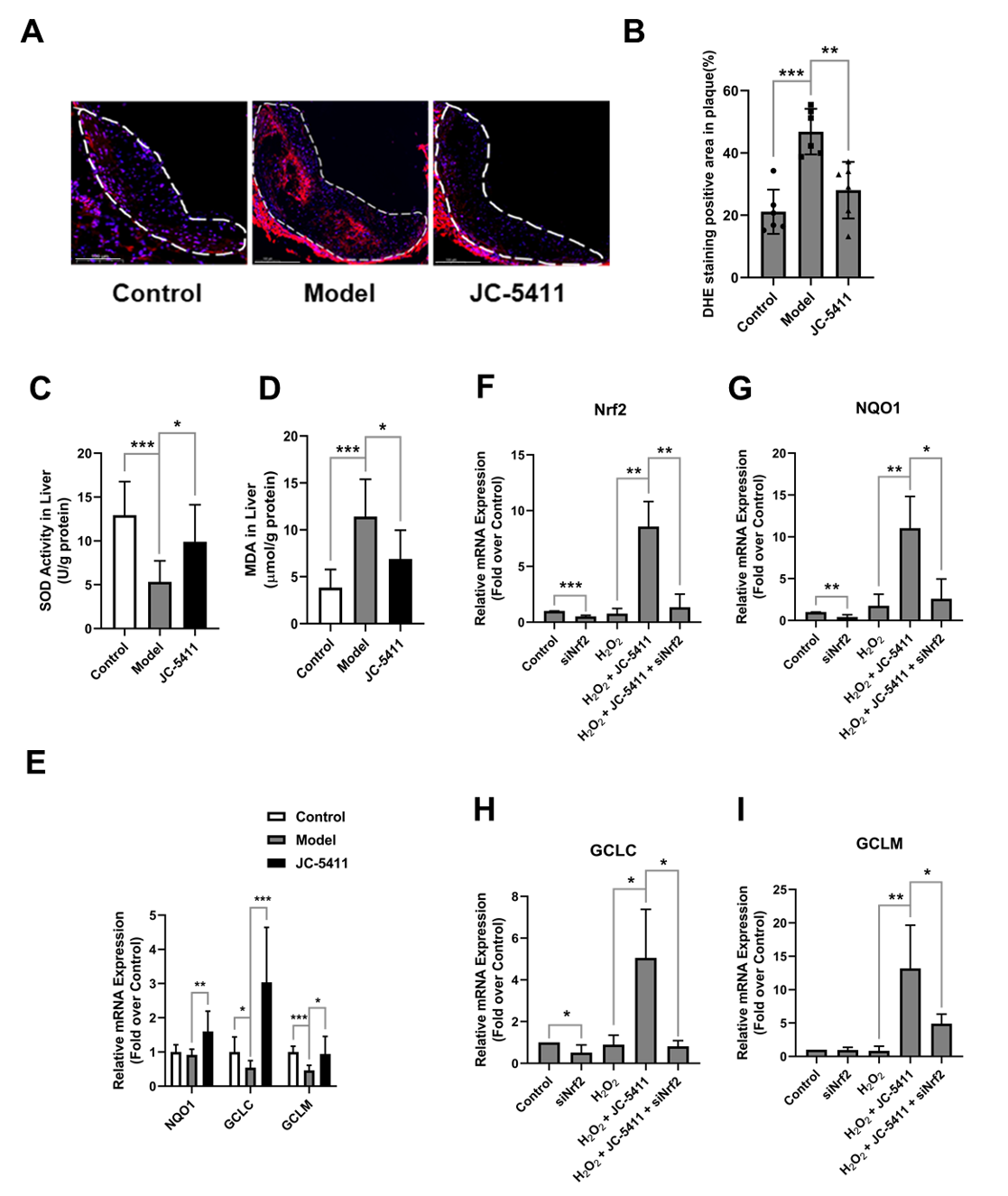

Supplement: Supplementary file 4 [file image4.tif]
